# Guide Tip Damage Due to Rotablation

**DOI:** 10.1016/j.jaccas.2025.105347

**Published:** 2025-09-10

**Authors:** Satoshi Taya, Masatoki Yoshida, Kentaro Ejiri, Hironobu Toda, Kazufumi Nakamura, Hiroshi Morita, Shinsuke Yuasa

**Affiliations:** a Department of Cardiovascular Medicine, Okayama University Graduate School of Medicine, Dentistry and Pharmaceutical Science, Okayama, Japan; b Department of Cardiovascular Medicine, Okayama University Faculty of Medicine, Dentistry and Pharmaceutical Sciences, Okayama, Japan

**Keywords:** complication, coronary angiography, imaging

## Abstract

**Background:**

The rotational atherectomy system can effectively debulk calcified coronary lesions. However, rare complications specific to that system have been reported.

**Case Summary:**

A 77-year-old man with a heavily calcified lesion in the right coronary artery (RCA) ostium underwent percutaneous coronary intervention in an 8-F system. During the procedure, rotablation with a 2.25-mm burr was required. After the percutaneous coronary intervention, partial loss of the tip of the guide was observed. He had no clinical sequelae despite the presumed retained catheter material in his body.

**Discussion:**

Although insufficient guide coaxiality has been suggested as the primary cause of guide tip fracture during RCA ostial ablation, other factors may have contributed: the application of force to the tip and a small difference in size between the guide and the burr.

**Take-Home Message:**

When ablating RCA ostial lesions, positioning the burr platform outside the guide may help prevent similar complications in future cases.

## Visual Summary

**Figure:**
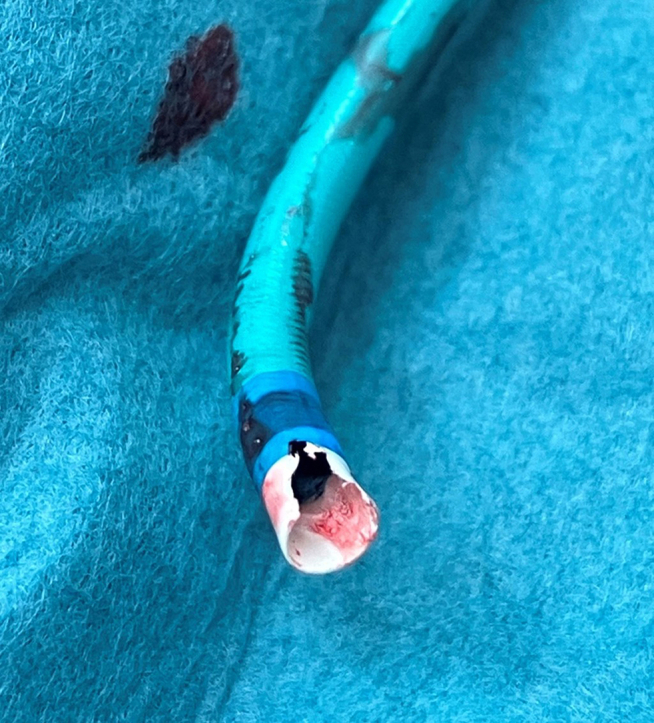

## History of Presentation

A 77-year-old man was readmitted for percutaneous coronary intervention (PCI) of a lesion at the right coronary artery (RCA) ostium 1 month after undergoing PCI for unstable angina pectoris due to a culprit lesion in the left anterior descending (LAD) coronary artery.

**Take-Home Messages**

• Rotablation of RCA ostial lesions carries the risk of poor device stability and increased procedural risk, including breakage of the tip of the guide.
• When ablating RCA ostial lesions, it is preferable to dislodge the guide from the RCA and position the burr platform out of the guide as much as possible.

## Past Medical History

He had severe renal dysfunction (estimated glomerular filtration rate 22.4 mL/min/1.73 m^2^) and also had a history of dyslipidemia, hypertension, and diabetes mellitus, which was being treated with insulin injections. He had smoked 20 cigarettes a day for 58 years.

## Differential Diagnosis

A significant lesion at the RCA ostium was suspected.

## Investigations

**Figure 1:**
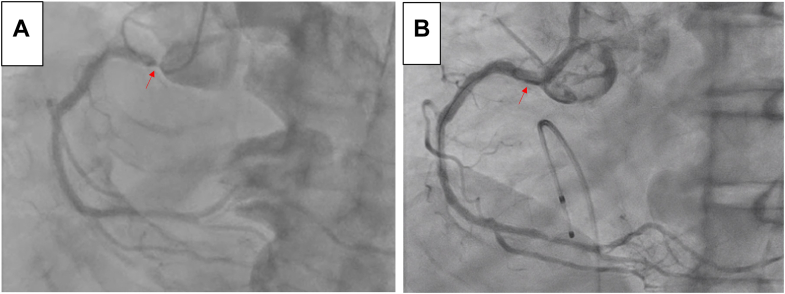
Coronary Angiography of the Right Coronary Artery (A) Before percutaneous coronary intervention (PCI). The lesion was at the ostium of the right coronary artery (red arrow). A near contrast shot was taken because the 4-F diagnostic catheter was wedged into the coronary artery. (B) After PCI. The lesion was well dilated (red arrow). There was no vessel rupture or delay in blood flow. A temporary pacemaker was placed in the right ventricle.

**Figure 2:**
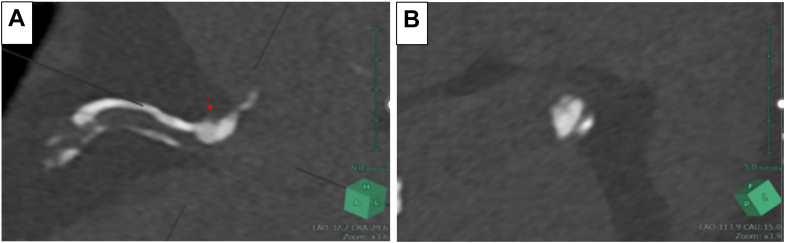
Thin-Slice Computed Tomography Image (A) The arrow indicates the thick calcified lesion at the ceiling of the right coronary artery ostium. (B) A short-axis view of the lesion shows a slit-like lumen wedged between calcified plaques.

At his previous admission for emergency PCI for the LAD lesion, coronary angiography of the RCA had revealed the present lesion (Figure 1A). During coronary angiography with a 4-F diagnostic catheter, the catheter was wedged into the ostial lesion of the RCA. Echocardiography showed a slight decrease in inferior wall motion, although his overall left ventricular ejection fraction was preserved at approximately 50%. In addition, considering his stage 4 renal dysfunction, a noncontrast cardiac computed tomography scan was performed, and it revealed a thick calcified lesion, up to 4.7 mm thick, at the RCA ostium (Figure 2).

## Management

Our heart team assessed that PCI was more suitable than coronary artery bypass surgery for the lesion because it was one residual lesion after PCI for an LAD culprit unstable angina pectoris. We considered that the RCA lesion could be modified with a rotational atherectomy device, and the patient agreed to PCI after being fully informed of the risks associated with the device.

**Figure 3:**
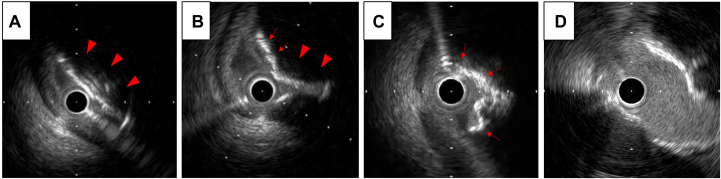
Intravascular Ultrasonography Images (A) Intravascular ultrasonography (IVUS) showed a nodular calcification on the upper side of the right coronary artery (RCA) ostium (arrowheads) and compression of the lumen by the plaque. (B) After the rotablation with a 2-mm burr, a considerable number of calcified nodules remained (arrowheads), although the lumen had widened partially (arrows). (C) After rotablation with a 2.25-mm burr, the calcified lesion in the RCA ostium was relatively evenly shaved (red arrows). (D) After the percutaneous coronary intervention, adequate dilation of the lesion was confirmed by IVUS.

PCI for the RCA lesion was performed via the femoral artery approach with an 8-F system. We selected an 8-F Launcher Judkins right 4 (JR4) guide (Medtronic) and crossed the lesion with an SION guidewire (Asahi Intecc). Intravascular ultrasound (IVUS) AltaView (Terumo) showed a nodular calcification on the upper side of the RCA ostium (Figure 3A). The calcification was so thick that we thought the lesion would be difficult to dilate with a balloon. Therefore, we decided to debulk it with a rotational atherectomy system device, rotablation. First, preparing for bradycardia during ablation, a temporary pacemaker was inserted into the right ventricle via the right femoral vein. Next, we changed the guidewire from SION to ROTAWIRE Extra Support (Boston Scientific), which is a stiffer wire with superior pushability and is considered effective in heavily calcified proximal lesions. We started the ablation with a 2-mm burr at 180,000 rotations per minute (rpm).

After the burr had passed through the lesion successfully, a considerable amount of nodular calcification remained, although the lumen had partially widened as shown by IVUS (Figure 3B). We therefore decided to increase the burr size to 2.25 mm and ablated again. To ensure that the wire was biased against the calcified lesion at the ceiling of the RCA ostium, we kept adding tension to pull up the guide during the ablation.

Throughout the procedure, the burr platform remained in the guide. When the burr crossed the lesion, there was some resistance and a decrease in rotational speed by 38,000 rpm. Consequently, transient ST-segment elevation in leads II, III, and aVF was observed. Fortunately, the patient did not experience any chest pain or hemodynamic changes, and no contrast delay was observed. After confirming adequate debulking of the lesion by IVUS (Figure 3C), we performed predilatation with a 3.25-mm cutting balloon, followed by a 3.5-mm paclitaxel-coated balloon. Finally, we could get a good final result with both angiography and IVUS (Figures 1B and 3D).

**Figure 4:**
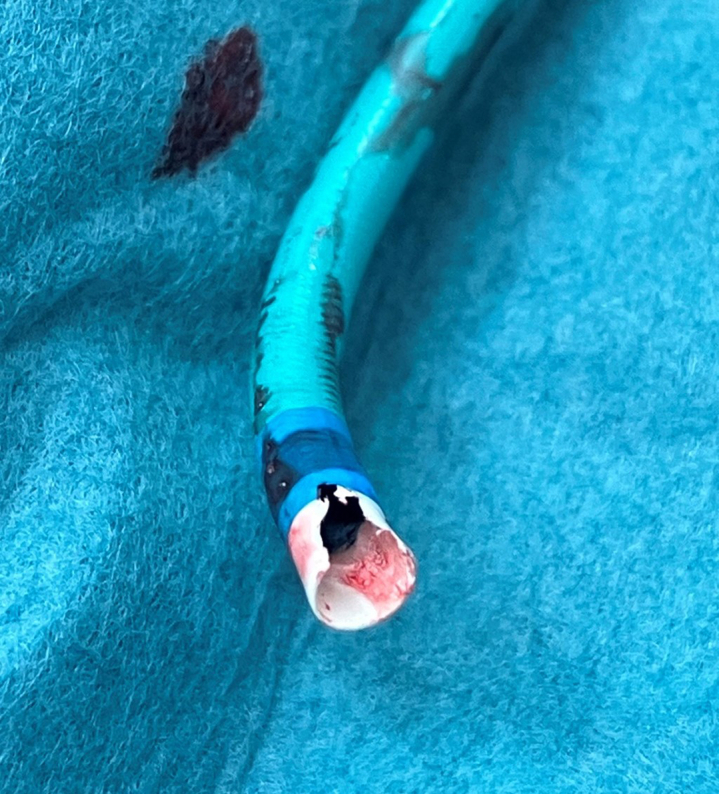
The Damaged Tip of the Guide Approximately a quarter of the circumference of the tip was damaged after percutaneous coronary intervention.

It was only after the catheter was removed from the body that we identified a substantial loss of the tip of the guide. Approximately a quarter of the circumference of the tip was damaged (Figure 4). There were no abnormal neurologic findings to suggest cerebral embolism. Cardiac computed tomography performed the day after PCI showed no obvious artifact in the coronary artery and myocardium. Blood tests indicated normal myocardial enzyme levels, and there were no ST-segment changes on electrocardiography, suggesting no coronary microembolism.

## Outcome and Follow-Up

He was discharged on the second postoperative day without any clinical sequelae, although there was probably some guide catheter material still in his body. Three months later, follow-up echocardiography showed normalization of inferior wall motion.

## Discussion

High-risk calcified lesions are treated with various innovations during PCI, including the use of rotablation.^1^, ^2^, ^3^ However, rotablation of RCA ostial lesions is known to be associated with poor device stability and increased procedural risk.^4^ Hurt et al^5^ published a case in 2016, in which the tip of the guide was similarly fractured during rotablation of a calcified lesion at the RCA ostium. In that case, the guide was a 7-F Amplatzer left 1 (inner diameter 2.05 mm) and the burr size was 1.50 mm. The authors concluded that the reason for the tip breakage was friction at the tip caused by the force exerted by the burr against the guide wall due to insufficient coaxiality. However, on the basis of our experience in the present case, we hypothesize several other factors in addition to insufficient coaxiality, as described below.

**Figure 5:**
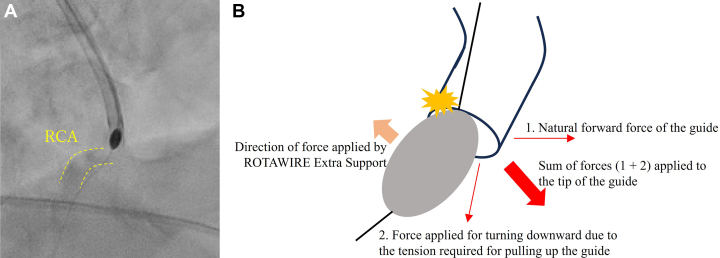
The Forces Applied to the Tip of the Guide (A) Right anterior oblique cranial view obtained by fluoroscopy showing the position of the guide, rota burr, and right coronary artery (RCA) (dashed line). (B) Schematic showing the sum and direction of the forces applied to the guide. The net force likely resulted in the burr being pressed against the upper side of the tip of the guide (yellow damage mark).

First, in terms of the burr force applied between the guide and the RCA ostium, the tip of the guide had an inherent tendency to rotate anteriorly, and when the catheter was pulled up, the tip tended to face downward. Therefore, the total force applied to the tip was in the forward-downward direction. On the other hand, the course through which the burr would pass was straightened by the ROTAWIRE Extra Support (Boston Scientific), a supportive guidewire, and the force applied to the burr was in the upward direction. This resulted in the burr pressing against the upper side of the tip of the guide, which likely damaged that area (Figures 5A and 5B).

**Figure 6:**
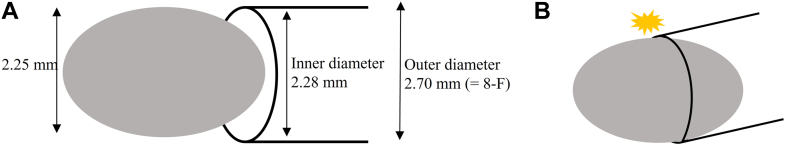
Comparison of Sizes of the Burr and the Guide (A) The sizes of the 2.25-mm rota burr and the inner and outer diameters of the 8-F guide were compared. There was almost no difference between the size of the rota burr and the inner diameter of the guide. (B) The burr tended to get stuck, leading to pressure on the tip of the guide because of the slightly oblique relationship between the guide and the burr.

The second issue is the size of guides and rota burrs. In this case, because we had anticipated the possibility of using a 2.25-mm rota burr, the largest burr size currently available in Japan, we adopted an 8-F guide for PCI via the femoral artery.^6^ We should mention that we could only use an 8-F guide when ablating with a 2.25-mm rota burr in Japan, because the guides in 9-F or greater sizes for PCI are not currently available in our country. An 8-F guide has an outer diameter of 2.70 mm and an inner diameter of 2.28 mm, which would barely allow a 2.25-mm burr to pass through (Figure 6A). Therefore, there were concerns that the burr could get stuck and cause pressure on the tip of the guide only if the burr and guide were at slightly different angles (Figure 6B). This situation might have contributed to the guide erosion.

**Figure 7:**
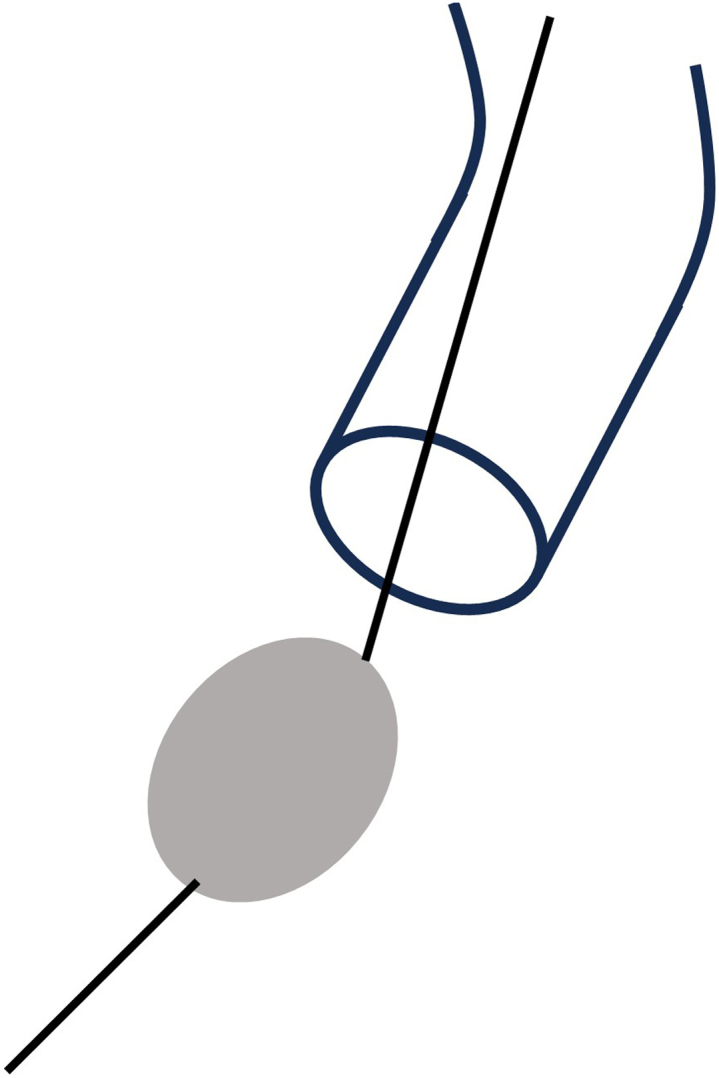
Disengaging the Guide Outside of the RCA Ostium The guide was dislodged from the RCA, and the burr platform was placed out of the guide to prevent damage to the tip of the guide. RCA = right coronary artery.

The factors mentioned so far are all based on the assumption that the burr platform was inside the guide and that the guide was in close proximity to the RCA ostium, such that the burr was sandwiched between the guide and the RCA ostium during ablation. Therefore, it is essential to avoid such conditions as much as possible, such as by disengaging the guide outside of the RCA ostium (Figure 7).

The lessons to be learned from this case are as follows. When ablating RCA ostial lesions:

1. It is preferable to dislodge the guide from the RCA and place the burr platform outside the guide.
2. If the guide cannot be dislodged from the coronary artery and the platform is still in the guide because of the need for a strong backup force or for any other reason, ablation should be performed carefully. Specifically, the duration of each ablation should be kept to a minimum. If resistance is encountered as the burr passes through the lesion, the burr should not be pushed with excessive force.

Despite these measures, if there is still a significant decrease in rotation speed, it is essential to check for coronary blood flow or contrast leakage around the tip, which might indicate tip damage.

## Conclusions

Rotablation of RCA ostial lesions requires more careful ablation because of the potential for breakage of the tip of the guide. The first steps to take when ablating such lesions are dislodgement of the guide and positioning of the burr platform away from the RCA ostium.

## Funding Support and Author Disclosures

The authors have reported that they have no relationships relevant to the contents of this paper to disclose.
